# Clinically significant chronic liver disease in people with Type 2 diabetes: the Edinburgh Type 2 Diabetes Study

**DOI:** 10.1093/qjmed/hcv191

**Published:** 2015-10-09

**Authors:** J.R. Morling, J.A. Fallowfield, I.N. Guha, R.M. Williamson, M. Ali, S. Glancy, M.W.J. Strachan, J.F. Price

**Affiliations:** ^1^From the Centre for Population Health Sciences, University of Edinburgh, Old Medical Buildings, Teviot Place, Edinburgh EH8 9AG, UK; 2 Queen’s Medical Research Institute, University of Edinburgh, Edinburgh, UK; 3 NIHR Nottingham Digestive Diseases Biomedical Research Unit, University of Nottingham, Nottingham, UK; 4 Department of Metabolic Medicine, Edinburgh, UK; 5 Department of Radiology, Western General Hospital, Edinburgh, UK

## Abstract

**Background:** Type 2 diabetes is an independent risk factor for chronic liver disease, however disease burden estimates and knowledge of prognostic indicators are lacking in community populations.

**Aims:** To describe the prevalence and incidence of clinically significant chronic liver disease amongst community-based older people with Type 2 diabetes and to determine risk factors which might assist in discriminating patients with unknown prevalent or incident disease.

**Design:** Prospective cohort study.

**Methods:** Nine hundred and thirty-nine participants in the Edinburgh Type 2 Diabetes Study underwent investigation including liver ultrasound and non-invasive measures of non-alcoholic steatohepatitis (NASH), hepatic fibrosis and systemic inflammation. Over 6-years, cases of cirrhosis and hepatocellular carcinoma were collated from multiple sources.

**Results:** Eight patients had known prevalent disease with 13 further unknown cases identified (prevalence 2.2%) and 15 incident cases (IR 2.9/1000 person-years). Higher levels of systemic inflammation, NASH and hepatic fibrosis markers were associated with both unknown prevalent and incident clinically significant chronic liver disease (all *P* < 0.001).

**Conclusions:** Our study investigations increased the known prevalence of clinically significant chronic liver disease by over 150%, confirming the suspicion of a large burden of undiagnosed disease. The disease incidence rate was lower than anticipated but still much higher than the general population rate. The ability to identify patients both with and at risk of developing clinically significant chronic liver disease allows for early intervention and clinical monitoring strategies. Ongoing work, with longer follow-up, including analysis of rates of liver function decline, will be used to define optimal risk prediction tools.

## Introduction

Chronic liver disease (CLD) due to non-alcoholic fatty liver disease (NAFLD) in community populations represents a major challenge for general practitioners and a growing burden for healthcare services.^1^ Of the 25% of the UK population now categorized as obese, most will have NAFLD^2^ and ∼10% of these people have been diagnosed in community studies to have evidence of advanced liver fibrosis that leads to cirrhosis.^3^ Of patients with cirrhosis (all cause), 5–20% will develop hepatocellular carcinoma (HCC) in 5 years.^4^ Type 2 diabetes, which is increasing in frequency in parallel with obesity, is strongly associated with NAFLD but data on the progression to cirrhosis and HCC in community-based patients with diabetes is limited.

Three major independent reports have highlighted the need for the early detection of liver disease.^2^,^^5^^,^^6^^ The ability to identify patients with and at risk of developing clinically significant CLD (CS-CLD) would promote early intervention strategies and guide clinical follow-up, ensuring timely detection of cirrhosis where assimilation into surveillance programmes for HCC and varices as well as screening for cardiovascular disease has been shown to improve patient outcomes. Despite this, the existing diagnostic pathways for detection and onward referral of suspected CLD are based on traditional liver enzyme tests which lack accuracy and contribute to late diagnosis.

Using a population-based cohort of ∼1000 patients with Type 2 diabetes (The Edinburgh Type 2 Diabetes Study (ET2DS)^7^) we employed an extensive screening programme (including liver ultrasound and non-invasive measures of non-alcoholic steatohepatitis [NASH], hepatic fibrosis and systemic inflammation) for clinically significant liver disease to investigate the true burden of disease. Over 6-years, cases of cirrhosis, HCC and gastro-oesophageal varices were collated from multiple sources.

Within the diabetic population annual liver enzyme checks are commonly performed, but there are no clear and consistent national guidelines on their application and interpretation, particularly in the community setting. Despite this, they remain the main trigger for referral to specialist liver clinics. Using standard liver enzyme assessment and liver ultrasound scan (USS) to diagnose hepatic steatosis, we identified patients at potential risk of CS-CLD and followed them up prospectively to collect data on clinical outcomes.

Our aim was to describe the prevalence and incidence of CS-CLD amongst older people with Type 2 diabetes and to determine whether the detection of abnormal liver enzyme levels and/or the presence of hepatic steatosis on USS in people with Type 2 diabetes might assist in discriminating those patients who subsequently go on to develop CS-CLD. Finally, we investigated a wide range of potential risk factors and biomarkers, including complications of diabetes and markers of advanced CLD, which might be useful in identifying at-risk subjects.

## Methods

### The Edinburgh Type 2 Diabetes Study

Full methods of the ET2DS have been published elsewhere.^7^ Briefly, in 2006/7 patients aged 60–75 years with Type 2 diabetes were randomly selected from the Lothian Diabetes Register (LDR), a comprehensive register of patients with diabetes living in Lothian, Scotland, and invited to participate in the ET2DS.

One thousand and sixty-six men and women attended a baseline research clinic and were enrolled into the study. This study population has been shown to be largely representative of all patients randomly selected from the LDR and therefore of the target population of older men and women with Type 2 diabetes living in the general population.^8^

All survivors were invited to re-attend for clinical and liver investigation at Year 1 (2007/8) and at Year 4 (2010/11) with 939 (dead *n* = 15, unable to contact *n* = 19, unwilling/unable *n* = 93) and 831 study participants attending, respectively.

### Clinical and laboratory examination

Details of all the research clinics held at the Wellcome Trust Clinical Research Facility, Western General Hospital, Edinburgh, UK have been described previously.^7^,^^9^^ In brief, fasting venous blood sampling, physical examination and abdominal USS were undertaken.

A liver screen (including viral hepatitis serology, alpha-feto protein [AFP], liver autoantibodies, serum ferritin) was undertaken if the patient met the criteria for referral to specialist Hepatology services (see later) or if hepatic steatosis was present on USS and the participant had never previously had a liver screen.

All biochemical analytes were measured at the time of clinic attendance using a Vitros Fusion chemistry system (Ortho Clinical Diagnostics, Bucks, UK) at the Western General Hospital (Edinburgh, UK).

### Biomarkers of liver injury

A wide range of hepatic biomarkers were assessed including those indicating non-specific liver injury (liver enzymes: alanine aminotransferase [ALT], aspartate aminotransferase [AST] and gamma-glutamyl aminotransferase [GGT]), steatosis (USS-graded liver fat^10^), NASH (cytokeratin-18 [CK18]), surrogate measures of advanced portal hypertension (spleen size and platelet count) and liver fibrosis (AST to Platelet Ratio Index [APRI],^11^ AST:ALT ratio, Enhanced Liver Fibrosis [ELF] score,^12^ Fibrosis-4 score [FIB-4],^13^ hyaluronic acid [HA] and NAFLD Fibrosis Score [NFS]^14^).

CK18 and ELF were measured in stored serum samples (−80^°^C) as previously described.^15^

Abnormal liver enzymes were defined as: i) abnormal—greater than the upper limit of normal (ULN)—ALT >50 U/l, AST >45 U/l, GGT >55 U/l; ii) highly abnormal—greater than 2 × ULN for ALT, AST, GGT and iii) greater than recently proposed sex specific cut-offs for ALT—males >30 U/l, females >19 U/l.^16^

Serum marker panels for hepatic fibrosis were calculated as in original publications.

### Identification of liver disease

**Figure 1.**
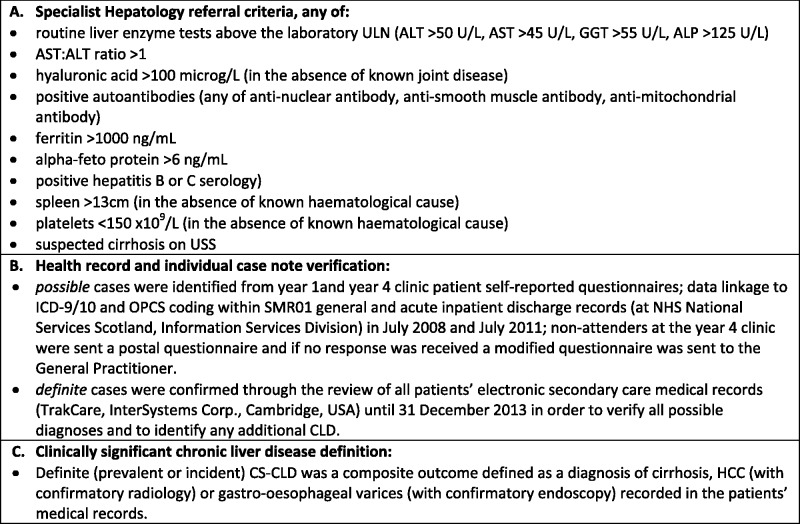
Identification of liver disease. ALP, alkaline phosphatase; ALT, alanine aminotransferase; AST, aspartate aminotransferase; CLD, chronic liver disease; CS-CLD, clinically significant chronic liver disease; GGT, gamma-glutamyl transferase; HCC, hepatocellular carcinoma; USS, ultrasound scan. Alcohol excess was defined as females >14 units/week, males >21 unis/week or patient disclosed history of a current or prior alcohol problem. Use of potentially hepatotoxic medication was defined as the use of non-topical glucocorticoids for >1 week, isoniazid, methotrexate, amiodarone or tamoxifen within the 6 months prior to the Year 1 clinic. Strongly positive autoantibodies were defined as ASMA titre >1:160 or AMA titre >1:40.

Identification of liver disease is described in Figure 1. Definite (prevalent or incident) CS-CLD was a composite outcome defined as a diagnosis of cirrhosis, HCC (with confirmatory radiology) or gastro-oesophageal varices (with confirmatory endoscopy) recorded in the patients’ medical records. Research study referral criteria for referral to specialist Hepatology services were designed in conjunction with an experienced consultant Hepatologist (Figure 1A), prevalent and incident CS-CLD was identified using a two-stage process across multiple sources (Figure 1B) and CS-CLD was defined as in Figure 1C.

### Data analysis

Data were analysed using SPSS v19.0 (SPSS Inc., IL). Variables that were not normally distributed were, where necessary, transformed and analysed on a logarithmic scale.

We describe the prevalence (both known and unknown) at the start of the study and subsequent incident CS-CLD. For initial analyses, all participants without known prevalent CS-CLD were included.

Exploratory analysis of potential risk factors and biomarkers associated with the development of advanced liver disease was undertaken using: patient characteristics, diabetes history and treatment, metabolic variables, established risk factors for CLD, markers of liver injury (non-specific, steatosis, NASH, advanced portal hypertension and liver fibrosis), and markers of systemic inflammation. Cox regression was used to investigate the association of potential risk factors with CS-CLD.

Ethical approval was obtained from the Lothian Research Ethics Committee and all subjects gave written informed consent.

## Results

### Subject characteristics

**Figure 2.**
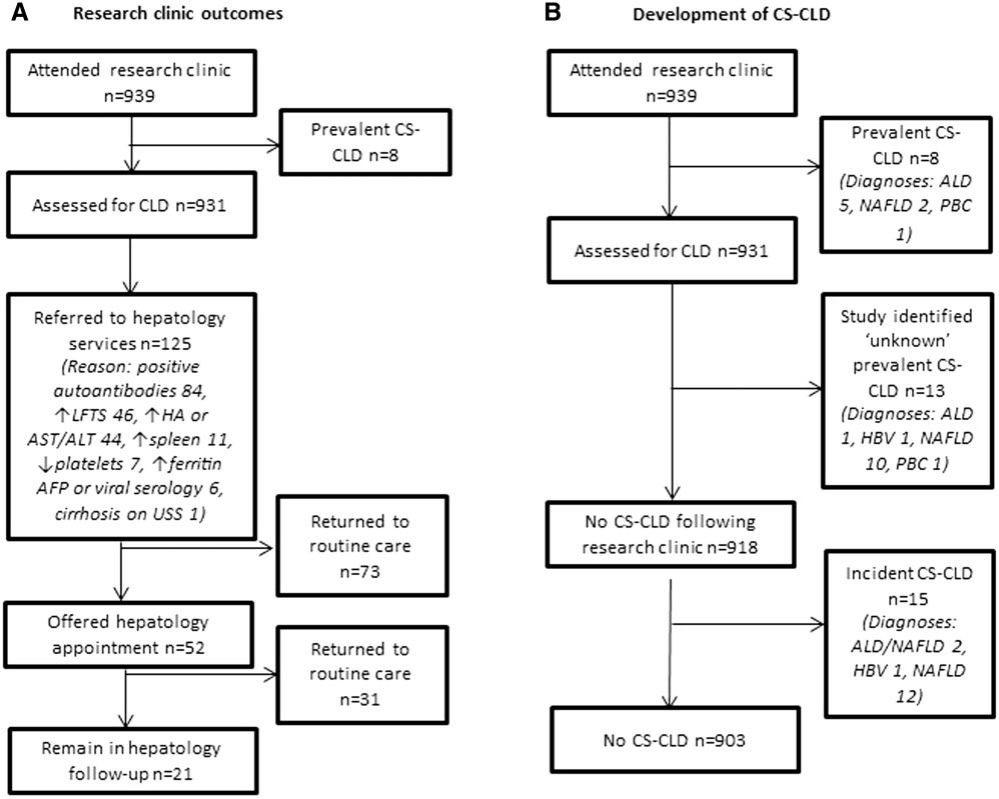
Patient flowcahrts. AFP, alpha-feto protein; ALD, alcoholic liver disease; ALT, alanine aminotransferase; AST, aspartate aminotransferase; CS-CLD, clinically significant chronic liver disease; HA, hyaluronic acid; HBV, hepatitis B virus; LFTs, liver function tests; NAFLD, non-alcoholic fatty liver disease; PBC, primary biliary cirrhosis.

Participant attendance is shown in Figure 2. Clinical follow-up data was available for all 939 Year 1 clinic attendees. Characteristics of the study population are shown in Table 1.

**Table 1.** Study population^a^

		Study population ***n***=923	Met Hepatology referral criteria ***n***=125	*P [****vs***.*not referred]*	Seen by Hepatology services ***n***=52	*P [****vs.****not seen]*
Age, years		68.9 (4.2)	69.3 (4.3)	*0.224*	69.3 (4.6)	*0.423*
Sex, % male		52.3% (483)	40.8 (51)	*0.007*	40.4 (21)	*0.087*
SIMD quintile, %	I	11.4 (105)	9.6 (12)	*0.041*	9.6 (5)	*0.031*
	V	34.3 (317)	31.2 (39)		25.0 (13)	
Random glucose, mmol/l		6.89 (2.3)	7.60 (3.0)	*0.003*	7.93 (3.3)	*0.021*
HbA1c, %		7.20 (1.1)	7.37 (1.2)	*0.046*	7.69 (1.3)	*0.008*
HbA1c, mmol/mol		55.2 (11.7)	57.1 (12.7)		60.5 (14.6)	
Duration of diabetes, % <5 years		25.7 (235)	20.0 (25)	*0.124*	17.3 (9)	*0.191*
Diabetes treatment:	Diet, %	19.5 (180)	19.2 (24)	*1.000*	19.2 (10)	*1.000*
	OAHA, %	64.8 (598)	63.2 (79)	*0.688*	59.6 (31)	*0.456*
	Insulin, %	15.7 (145)	17.6 (22)	*0.511*	21.2 (11)	*0.245*
Retinopathy, %	Mild	27.9 (254)	24.2 (30)	*0.182*	25.0 (13)	*0.035*
	Moderate/severe	4.4 (40)	7.2 (9)		11.5 (6)	
Chronic kidney disease^b^, %		19.6 (179)	22.8 (28)	*0.393*	27.5 (14)	*0.149*
Cardiovascular disease^c^, %		36.9 (338)	37.6 (47)	*0.842*	38.5 (20)	*0.769*
BMI, kg/m^2^		31.3 (5.7)	31.7 (6.1)	*0.411*	32.0 (6.6)	*0.414*
Total cholesterol, mmol/l		4.4 (0.8)	4.3 (0.8)	*0.021*	4.1 (0.8)	*0.690*
Triglycerides, mmol/l		1.66 (0.9)	1.74 (1.0)	*0.305*	1.50 (0.6)	*0.073*
sBP, mmHg		138.2 (18.5)	140.6 (18.6)	*0.115*	138.7 (17.1)	*0.845*
Known risk factor for liver disease^d^, %		20.9 (193)	31.2 (39)	*0.004*	34.6 (18)	*0.021*
Alcohol excess^e^, %		12.8 (118)	20.8 (26)	*0.006*	21.2 (11)	*0.084*
Hepatotoxic medication^f^, %		9.2 (6)	10.4 (13)	*0.618*	11.5 (6)	*0.467*
Positive autoantibodies^g^, %		0.7 (6)	3.2 (4)	*0.005*	3.8 (2)	*0.046*

^a^Values are mean (SD), median (IQR) or % (***n***). BMI, body mass index; IQR, inter-quartile range; OAHA, oral anti-hyperglycaemic agent; sBP, systolic blood pressure; SD, standard deviation; SIMD, Scottish Index of Multiple Deprivation.

^b^Defined as estimated glomerular filtration rate <60 ml/min.

^c^Defined as of myocardial infarction, angina, coronary intervention, intermittent claudication, peripheral artery intervention, stroke, transient ischaemic attack or carotid endarterectomy.

^d^Defined as any of d–f later.

^e^Defined as females >14 units/week, males >21 unis/week or patient disclosed history of a current or prior alcohol problem.

^f^Defined as the use of (non-topical) glucocorticoids for >1 week, isoniazid, methotrexate, amiodarone or tamoxifen within the 6 months prior to the liver assessment.

^g^Defined as ASMA titre >1:160 or AMA titre >1:40.

### **Prevalent **CS-CLD

Eight patients had known prevalent CS-CLD and were excluded from the investigation of unknown prevalent disease (*n* = 931, Figure 2A).^17^

Following the Year 1 investigations, 13.4% (125/931) of participants met the research study protocol criteria as high risk for the presence of liver disease and were referred to Hepatology. Risk factors for referral are shown in Table 1.

Of these, 52 (41.6%) were offered an appointment at Hepatology clinic and the remainder returned to standard care after triage by a consultant Hepatologist. The majority of those not seen had low level titres of positive autoantibodies (≥1:40) or isolated mildly elevated liver enzymes only (<2 × ULN).

Of those referrals seen, 31 (59.6%) were immediately discharged following initial assessment on the basis of having either simple fatty liver with low risk for the presence of significant liver fibrosis (according to non-invasive marker criteria) and/or false positive indicators of CS-CLD (e.g. platelets <150 × 10^9^/l but no portal hypertension) as determined by a consultant Hepatologist. Twenty-one participants (40.4) remain under active Hepatology follow-up.

As a consequence of the Year 1 investigations, 13 (1.4%) patients were diagnosed with previously unknown prevalent CS-CLD (including one HCC, the remainder cirrhosis). The reasons for their initial referral were wide-ranging.

Risk factors associated with unknown prevalent cases were elevated liver enzymes (but not ALT), inflammatory markers, markers of NASH and hepatic fibrosis (all *P* < 0.01, Tables 2 and 3).

**Table 2.** Potential demographic, diabetes and metabolic risk factors for the development of CS-CLD^a^

		No CS-CLD ***n***=903	Unknown prevalent CS-CLD ***n***=13	OR (95% CI) for unknown prevalent CS-CLD	*P*	Incident CS-CLD ***n***=15	HR (95% CI) for incident CS-CLD	*P*
Age, years		68.9 (4.2)	69.5 (4.9)	1.04 (0.91,1.18)	*0.600*	69.8 (4.1)	1.07 (0.94,1.21)	*0.306*
Sex, % male		52.5 (474)	30.8 (4)	2.49 (0.76,8.13)	*0.132*	40.0 (6)	1.93 (0.69,5.45)	*0.213*
SIMD quintile:	I, %	11.2 (101)	7.7 (1)			33.3 (5)	Ref	
	V,%	34.9 (315)	0 (0)			26.7 (4)	0.26 (0.07,0.97)	*0.044*
Duration of diabetes >5 years, %		73.7 (660)	76.9 (10)	1.19 (0.32,4.35)	*0.796*	93.3 (14)	5.30 (0.70,40.34)	*0.107*
Fasting glucose, mmol/l		6.87 (2.3)	7.53(3.2)	1.11 (0.91,1.35)	*0.301*	6.98 (2.8)	1.21 (0.78,1.88)	*0.405*
HbA1c,	%	7.19 (1.1)	7.34 (0.8)	1.13 (0.70,1.84)	*0.615*	7.53 (1.5)	1.02 (0.98,1.06)	*0.405*
	mmol/mol	55.1 (11.6)	56.7 (8.3)	1.01 (0.97,1.06)		58.8 (15.9)		
Diabetes treatment:	Diet, %	19.7 (178)	15.4 (2)	Ref		6.7 (1)	Ref	
	OAHA, %	65.1 (588)	46.2 (6)	1.06 (0.22,5.15)	*0.943*	53.3 (8)	2.50 (0.31,20.0)	*0.388*
	Insulin, %	15.2 (137)	69.2 (9)	2.60 (0.47,14.4)	*0.274*	40.0 (6)	9.08 (1.09,75.5)	*0.041*
BMI, kg/m^2^		31.2 (5.6)	33.4 (6.7)	1.06 (0.98,1.16)	*0.161*	34.8 (7.4)	1.09 (1.02,1.17)	*0.016*
Cholesterol, mmol/l		4.2 (0.8)	3.9 (0.5)	0.68 (0.32,1.43)	*0.310*	3.9 (0.8)	0.66 (0.33,1.34)	*0.248*
Triglycerides, mmol/l		1.67 (0.9)	1.25 (0.3)	0.39 (0.14,1.11)	*0.078*	1.46 (0.4)	0.70 (0.32,1.54)	*0.371*
CRP^b^, mg/l		1.69 (0.8–3.9)	2.72 (1.4–11.6)	1.83 (1.14,2.94)	*0.013*	3.98 (1.9–13.6)	1.66 (1.06,2.60)	*0.026*

^a^Values are % (***n***), ORs and HRs. CRP, C-reactive protein; CS-CLD, clinically significant chronic liver disease; HR, hazard ratio; OR, odds ratio; SD, standard deviation; SIMD, Scottish Index of Multiple Deprivation. CS-CLD defined as clinically significant disease: incident cirrhosis, HCC or gastro-oesophageal varices.

^b^Analysed on the Ln scale: ORs and HRs for a one unit increase in the ln of the risk factor.

**Table 3.** Potential liver injury related risk factors for the development of CS-CLD^a^

	No CS-CLD ***n***=903	Unknown prevalent CS-CLD ***n***=13	OR (95% CI) for unknown prevalent CS-CLD	*P*	Incident CS-CLD ***n***=15	HR (95% CI) for incident CS-CLD	*P*
ALT, U/l	33.3 (12.7)	39.0 (12.6)	1.03 (1.00–1.06)	*0.108*	44.4 (23.6)	1.04 (1.01,1.06)	*0.002*
ALT >50 U/l, %	7.8 (70)	23.1 (3)	3.56 (0.96,13.2)	*0.058*	40.0 (6)	6.69 (2.38,18.8)	*<0.001*
AST, U/l	30.0 (9.5)	49.7 (12.6)	1.09 (1.06–1.13)	*<0.001*	46.0 (23.7)	1.07 (1.04,1.09)	*<0.001*
AST >45 U/l, %	6.8 (61)	69.2 (9)	30.8 (9.22,102.9)	*<0.001*	40.0 (6)	8.30 (2.94,23.43)	*<0.001*
GGT^b^, U/l	16.0 (10.0–26.0)	55.0 (34.0–103.0)	5.35 (2.91,9.83)	*<0.001*	62.0 (21.5–185.0)	3.56 (2.17,5.83)	*<0.001*
GGT >55 U/l, %	7.9 (71)	69.2 (9)	26.2 (7.87,87.1)	*<0.001*	53.3 (8)	13.2 (4.79,36.6)	*<0.001*
CK18^b^, U/l	100.6 (76.2–135.5)	152.7 (143.1–207.9)	3.42 (1.59,7.34)	*0.002*	127.6 (83.4–586.5)	4.10 (2.08,8.06)	*<0.001*
Hepatic steatosis, %	56.8 (513)	46.2 (6)	0.65 (0.22,1.95)	*0.445*	80.0 (12)	2.93 (0.83,10.38)	*0.096*
APRI*	0.24 (0.19–0.32)	0.67 (0.35–0.92)	87.7 (20.8,369.6)	*<0.001*	0.39 (0.29–0.88)	20.4 (6.81,61.0)	*<0.001*
AST:ALT	0.95 (0.3)	1.33 (0.3)	2.55 (1.22,5.34)	*0.013*	1.10 (0.3)	1.56 (0.93,2.64)	*0.094*
AST:ALT ratio >1, %	35.7 (320)	76.9 (10)	6.00 (1.64,22.0)	*0.007*	60.0 (9)	3.67 (1.29,10.44)	*0.015*
ELF score	8.9 (0.8)	10.9 (1.0)	4.38 (2.20,8.70)	*<0.001*	10.2 (1.0)	1.64 (1.30,2.06)	*<0.001*
FIB4 score	1.50 (0.6)	3.96 (2.0)	7.81 (3.93,15.5)	*<0.001*	2.56 (1.3)	4.08 (2.71,6.15)	*<0.001*
HA^b^, micrg/l	50.6 (34.8–81.4)	220.3 (177.9–318.9)	16.4 (5.35,50.1)	*<0.001*	183.2 (78.9–230.6)	5.80 (2.60,13.0)	*<0.001*
NFS	−0.40 (1.1)	1.38 (1.5)	3.99 (2.36,6.73)	*<0.001*	0.74 (1.0)	2.18 (1.54,3.09)	*<0.001*
Spleen >13 cm, %	4.1 (37)	69.2 (9)	52.5 (15.5,178.5)	*<0.001*	0		
Platelets <150 x 10^9^/l, %	3.0 (27)	46.2 (6)	27.3 (8.59,86.6)	*<0.001*	14.3 (2)	4.36 (0.97,19.6)	*0.055*
Known risk factor for liver disease^c^, %	20.7 (187)	30.8 (4)	1.70 (0.52,5.59)	*0.488*	40.0 (6)	2.44 (0.87,6.85)	*0.091*
Alcohol excess^d^, %	12.6 (113)	23.1 (3)	2.09 (0.57,7.71)	*0.268*	20.0 (3)	1.63 (0.46,5.77)	*0.451*

^a^Values are % (***n***), ORs ratios and HRs. ALT, alanine aminotransferase; APRI, Aspartate to Platelet Ration Index; AST, aspartate aminotransferase; CK18, cytokeratin-18; CS-CLD, clinically significant chronic liver disease; ELF, Enhanced Liver Fibrosis panel; FIB4, Fibrosis-4 Score; GGT, gamma-glutamyl transferase; HA, hyaluronic acid; HR, hazard ratio; NFS, NAFLD Fibrosis Score; OR, odds ratio. CS-CLD defined as clinically significant disease: incident cirrhosis, HCC or gastro-oesophageal varices.

^b^Analysed on the Ln scale: ORs and HRs for a one unit increase in the ln of the risk factor.

^c^Defined as any of: alcohol excess (females >14 units/week, males >21 unis/week or patient disclosed history of a current or prior alcohol problem), hepatotoxic medication use (use of (non-topical) glucocorticoids for >1 week, isoniazid, methotrexate, amiodarone or tamoxifen within the 6 months prior to the liver assessment) or positive autoantibodies (ASMA titre >1:160 or AMA titre >1:40).

^d^Defined as females >14 units/week, males >21 unis/week or patient disclosed history of a current or prior alcohol problem.

Overall the prevalence of CS-CLD was 2.2% (known plus unknown).

### **Development of incident **CS-CLD

Nine hundred and eighteen patients (Figure 2B) did not have prevalent CS-CLD.

Over a mean follow-up period of 5.6 years (SD 1.0, total 5156 person-years) there were 15 incident cases of CS-CLD, IR 2.9/1000 person-years. These comprised cirrhosis *n* = 14 (2.7/1000 person-years), HCC *n* = 4 (0.8/1000 person-years) and gastro-oesophageal varices *n* = 5 (1.0/1000 person-years), with some patients having more than one complication.

Patients identified as potentially high risk after the Year 1 investigations were more likely to develop CS-CLD than those not thought to have liver disease (IR 10.9 vs. 1.8/1000 person-years, *P* = 0.001). However, of the incident 15 patients that developed CS-CLD less than half (*n* = 7, 46.7%) were identified as high risk by the extensive liver-related investigations at the Year 1 clinic. Of the incident HCC, only 1 (25.0%) occurred in a participant identified through our Year 1 investigations. The majority of cases of varices (*n* = 3, 60.0%) were identified through entry into an endoscopic surveillance programme.

Rates of incident CS-CLD were significantly higher in those: seen in the Hepatology clinic (IRR 18.7, 95%CI 6.5–54.0, *P* < 0.001), with abnormal liver enzymes (IRR 5.7, 95%CI 2.0–16.0, *P* = 0.001) and with very abnormal liver enzymes (IRR 16.3, 95%CI 5.2–50.9, *P* < 0.001). Individuals with hepatic steatosis or elevated liver enzymes as defined by the lower revised laboratory reference ranges had incidence rates of CS-CLD not significantly different to those without (IRR 2.9, *P* = 0.096 and IRR 1.2, *P* = 0.800, respectively).

Patients with abnormal liver enzymes at the Year 1 investigation were more likely to develop CS-CLD, (normal 7/776, 0.9%; abnormal 3/113, 2.7%; very abnormal 5/26, 19.2%; *P* < 0.001). In those developing CS-CLD, mean/median levels of liver enzymes were higher, however they remained broadly within normal laboratory limits for transaminases (ALT 44.4 U/l, AST 46.0 U/l), with median GGT levels above the ULN (median 62 vs. 16 U/l, *P* < 0.001) (Table 2), overall 47% of those with incident CS-CLD had normal liver enzymes (Table 3).

Tables 2 and 3 show the associations between potential risk factors and incident CS-CLD. Known causes of liver disease (including alcohol) were similar in both groups (*P* = 0.123). Higher levels of markers of systemic inflammation were associated with an increased incidence of CS-CLD (ln C-reactive protein hazard ratio (HR) 1.66 *P* = 0.026, ln IL-6 HR 2.86 *P* = 0.002, ln TNF-α HR 2.12 *P* = 0.017)

Nearly all patients (80.0%) with incident CS-CLD had hepatic steatosis at the start of the study, although this was not statistically significant. All of the continuous markers of NASH and hepatic fibrosis (except AST:ALT ratio) were higher in those with incident CS-CLD than in those who did not.

## Conclusions

We conducted the only large prospective community-based study of older patients with Type 2 diabetes. The prevalence of CS-CLD at baseline was 2.2% (0.9% diagnosed clinically prior to enrolment and 1.4% identified by study investigations). The incidence of CS-CLD was 1.4% over nearly 6 years (IR 2.9/1000 person-years).

Existing estimates of CS-CLD frequency are limited by reliance on confirmatory liver biopsy in secondary care populations or in community-based cohorts by the use of a single screening modality (e.g. FibroTest,^18^ transient elastography^19^) each with their own challenges; e.g. liver enzymes have low diagnostic accuracy, non-invasive fibrosis markers (e.g. ELF, NFS) have poor positive predictive values for later stages of CLD,^12^,^^14^^ ICD codes only identify hospitalized cases with decompensation and biopsy has marked ascertainment bias. Accordingly, in the general population, the prevalence of advanced fibrosis in NAFLD ranged from 0 to 12% in a single study depending on the markers used,^20^ and there is even greater discordance between different studies on CS-CLD prevalence. Therefore, we used a multimodal approach to comprehensively phenotype our cases and maximize detection CS-CLD.

Our findings support the suggestion that NAFLD is an under-diagnosed chronic disease^21^ despite patients with Type 2 diabetes having, as a minimum, annual clinical reviews including liver enzyme tests. Our multimodal diagnostic approach increased the prevalence by more than 150% through the diagnosis of unknown CS-CLD, of which almost 70% was attributable to NAFLD. However, it was a labour intensive process with 14% of the study participants referred to a consultant Hepatologist. In the majority of these patients, simple laboratory tests were abnormal (e.g. liver enzymes above the ULN, low platelet count) although, typically, values were only marginally elevated.

Reassuringly, despite a relatively high prevalence of uncomplicated NAFLD at Year 1 there were only a small number of patients who went on to develop incident CS-CLD after 6 years (2.9/1000 person-years). In 103 NAFLD patients followed by Adams *et al*.^22^ for a mean of 3.2 years, the fibrosis progression rate was 0.35 stages/year in those with diabetes. This is high compared to our cohort as it equates to a potential 2-stage advancement over the 6 year follow-up of our cohort in those with NAFLD. In NASH populations, 32% of subjects progressed fibrosis score over 3 years^23^ and 31% had progressive fibrosis,^24^ although no participant developed cirrhosis during 4 years of follow-up.

The only aspect of diabetes history to be associated with the development of CS-CLD was the use of insulin (HR 9.08). Hyperinsulinemia associated with T2DM is a risk factor for NASH, hepatic fibrosis and HCC. Insulin stimulates hepatic stellate cell proliferation and collagen synthesis *in vitro* and therefore, by extension, exogenous insulin therapy could promote liver fibrosis in vivo.^25^ Our data reinforces the known association between the presence of diabetes and increased the risk of HCC.^26^ Moreover, Scottish Cancer Registry data showed that within the whole Scottish population aged 60–75 the IR for liver-related cancer (including biliary) in 2012 was 0.3/1000 person-years,^27^ which is substantially lower than the rate for HCC alone in our cohort (0.8/1000 person-years).

Participants in the least deprived Scottish Index of Multiple Deprivation (SIMD) quintile had an incidence rate of CS-CLD 74% lower than those in the most deprived quintile. This unadjusted finding may represent confounding by other factors such as alcohol and obesity. Previous studies into the relationship between CLD and deprivation have found any associations were lost after adjustment for such risk factors.^28^

Critically, for clinical practice, the majority of incident CS-CLD had normal liver function tests with no steatosis. Whilst those with abnormal liver enzymes *per se* were more likely to develop CS-CLD, the mean levels of liver enzymes in those that did were still within the normal laboratory reference range. It may be that given the high prevalence of steatosis in the cohort that it may add little risk of CS-CLD beyond the risk conferred by Type 2 diabetes alone.

The main limitation to this study is the lack of liver biopsy, although this represents an imperfect ‘gold standard’ test for the diagnosis and staging of NAFLD and is impractical in community studies with a low prevalence of CS-CLD. Additionally, we would argue that our non-invasive liver assessment is more applicable to clinical practice and is unlikely to have missed CS-CLD due to the use of validated cut-offs and robust triage for referral to a consultant Hepatologist.

This work suggests that there is little benefit in performing liver USS of patients similar to the study cohort, where the finding of hepatic steatosis could be predicted and provides no indication of future disease progression.

For the first time, we have evaluated the utility of extensive but simple targeted investigation using routinely available clinical tests to identify those at high risk of both immediate and future CS-CLD. We plan to conduct longer term follow-up to capture future incident liver-related events. Of particular interest will be how the rate of change of potential biomarkers in earlier stages of CLD can be used to predict future development of CS-CLD.
